# Evaluation of the Healthy Living after Cancer text message-delivered, extended contact intervention using the RE-AIM framework

**DOI:** 10.1186/s12885-021-08806-4

**Published:** 2021-10-07

**Authors:** Jennifer R. Job, Elizabeth G. Eakin, Marina M. Reeves, Brianna S. Fjeldsoe

**Affiliations:** 1 Faculty of Medicine, School of Public Health, The University of Queensland, Brisbane, Australia; 2 CHSRI, The University of Queensland, RBWH, Level 8, Health Sciences Building, Herston, Q 4029 Australia

**Keywords:** Text messages, Extended contact, Diet, Physical activity, Weight, Maintenance, RE-AIM

## Abstract

**Background:**

Text message-delivered interventions have potential to prevent weight regain and maintain diet and physical activity behaviours through extending contact with participants following initial weight loss, lifestyle interventions. Using the RE-AIM Framework, this study evaluated the adoption, reach, implementation, effectiveness, and maintenance of an extended contact text-message intervention following the Healthy Living after Cancer (HLaC) program. HLaC was a 6-month, telephone-delivered intervention targeting healthy diet, physical activity and weight loss for adult cancer survivors, offered by Cancer Councils (CCs) in Australia.

**Methods:**

HLaC completers (*n* = 182) were offered extended contact via text messages for 6-months (HLaC+Txt). Text message content/frequency was individually tailored to participant’s preferences, ascertained through two telephone-tailoring interviews with CC staff. Adoption (HLaC+Txt uptake among eligible CCs), reach (uptake by HLaC completers) and implementation (intervention cost/length; text dose) were assessed. The effectiveness of extended contact relative to historic controls was quantified by pre-to-post HLaC+Txt changes in self-reported: weight, moderate-vigorous physical activity (MVPA), fruit and vegetable intake, fat and fibre behaviour. Maintenance, following 6-months of noncontact for the intervention cohort, was assessed for these same variables. Semi-structured interviews with CC staff and participants contextualised outcomes.

**Results:**

HLaC+Txt was adopted by all four CCs who had delivered HLaC. In total, 115 participants commenced HLaC+Txt, with reach ranging across CCs from 47 to 80% of eligible participants. The mean number of weeks participants received the text message intervention ranged across CCs from 18.5–22.2 weeks. Participants received (median, 25th,75th percentile) 83 (48, 119) texts, ranging across CCs from 40 to 112. The total cost of HLaC+Txt delivery was on average $AUD85.00/participant. No meaningful (*p* < 0.05) differences in self-reported outcomes were seen between HLaC+Txt and control cohorts. After 6-months no contact the intervention cohort had maintained weight, fruit intake, fat and fibre index scores relative to end of HLaC+Txt outcomes. Participants/CC staff perceived an important intervention component was maintaining accountability.

**Conclusions:**

While feasible to implement, HLaC+Txt was not effective in the short term. However, intervention effects during the non-contact period suggest the program supports longer term maintenance of weight and diet behaviour. Intervention delivery in this real-world context highlighted key considerations for future implementation.

**Trial registration:**

Australian and New Zealand Clinical Trials Registry (ANZCTR) - ACTRN12615000882527 (registered on 24/08/2015).

**Supplementary Information:**

The online version contains supplementary material available at 10.1186/s12885-021-08806-4.

## Background

Worldwide, there is a positive trend towards improved rates of survival for people diagnosed with breast, colorectal, prostrate, melanoma and endometrial cancer [1]. In Australia the overall cancer survival rate was 69% in 2011–2015, a substantial increase from 48% in 1984–1988 [2, 3]. For those diagnosed with the most prevalent cancers (i.e. breast, prostate, bowel), many are now more likely to die from causes other than cancer (i.e. cardiovascular disease) compared to other non-cancer survivor populations [4]. To reduce the risk of cancer recurrence and comorbidities, lifestyle modifications are recommended, including achieving a healthy weight, being physically active and increasing the intake of wholegrains, vegetables and fruit [3–6].

Lifestyle interventions for cancer survivors have been effective for supporting weight loss and achieving improvements in diet and physical activity behaviours [7–14]. However, as with interventions in the general population, maintaining dietary and physical activity behaviour changes following intervention completion remains a challenge [7, 10].

Extended contact interventions can provide continued support to prevent weight regain and to maintain the healthy physical activity and diet behaviours [15, 16]*,* with meta-analyses supporting their effectiveness [16–18]. Extended contact interventions are delivered after more intensive initial interventions and typically have a tapered intervention dose and a behavioural maintenance focus [9, 19–22]. Most extended contact intervention trials have implemented contact modalities that were used during the initial intervention contact, such as telephone support [9], group conference calls [22] and face-to-face groups with telephone or email support [20]. To date, two published extended contact interventions following a lifestyle intervention have been delivered by text message: one in a general adult population [23], and one among cancer survivors [19]. Both trials showed some promising results and have progressed into implementation trials, as a key benefit of utilizing text messages is that they offer a low cost, broad reach method of delivery of extended contact by community partners. However, literature on the implementation of extended contact interventions in community settings remains limited [24, 25].

Using the RE-AIM framework [26], this paper describes the outcomes of a text message-delivered, extended contact intervention for Healthy Living after Cancer (HLaC). HLaC was a 6-month, telephone-delivered health coaching intervention targeting physical activity, diet and weight loss in cancer survivors who had previously been treated with curative intent [27, 28]. The intervention was delivered by Cancer Councils (CCs) across Australia and evaluated in the context of a dissemination and implementation trial [27, 28]. Following completion of the HLaC program, clients were offered HLaC+Txt, a 6-month text message-delivered, extended contact intervention. It was hypothesised that, on average, those who had received HLaC+Txt would maintain or improve weight, diet and physical activity behaviour outcomes, while those who did not receive HLaC+Txt would regress towards baseline levels, resulting in a significant between cohort difference in intervention effect at the end of HLaC+Txt.

## Methods

### Study design

**Fig. 1:**
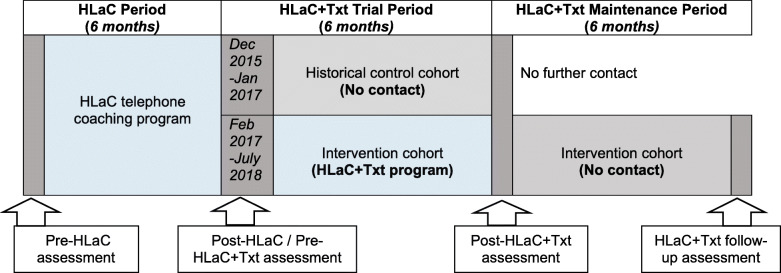
HLaC+Txt Trial Study Design

A historical control design was used to evaluate the addition of HLaC+Txt to the HLaC intervention. HLaC+Txt was compared to the standard HLaC protocol (i.e. no further intervention contact) (Fig. 1). Recruitment into HLaC commenced in June 2015 and participants completing the HLaC telephone-coaching program between December 2015 and January 2017 and who consented to completing an additional assessment (6-months after completing HLaC) formed the historical control cohort. All participants who completed HLaC between February 2017 and July 2018 and who owned a mobile telephone were invited to receive HLaC+Txt, with an additional follow-up evaluation 6 months after HLaC+Txt completion (Fig. 1). Ethical clearance was approved by the human research ethics committees of The University of Queensland (2,014,001,106/HREC 1407), the participating CCs and by referring clinical sites.

### Study context

The initial HLaC program was delivered via telephone with up to 12 calls from CC nurses/ allied health professionals and was supplemented with a printed Participant Workbook [27]. The coaches were experienced in cancer care and trained to deliver the HLaC program using motivational interviewing techniques [27, 28]. During HLaC, participants were guided to develop skills in behaviour change techniques for improving physical activity and dietary behaviours including: goal setting, self-monitoring, problem solving, identifying social support, stimulus control, positive self-talk and self-reward [29]. Four of the five CCs in Australia took up the initial HLaC program and 786 eligible cancer survivors participated in HLaC (88.7% overall uptake), with outcomes reported elsewhere [28].

### Recruitment

Participants eligible for HLaC were adults (18+ years) diagnosed with any form of localised cancer (non-metastatic) who had been treated with curative intent [28]. CC delivery staff recruited participants for the HLaC+Txt trial during their final HLaC coaching call or the post-HLaC assessment, and if participants agreed, recorded verbal or written consent was obtained.

### Intervention cohort treatment

As outlined in Table 1, HLaC+Txt participants received two tailoring interviews (initial and 12-weeks) and tailored text messages over 24 weeks. The content, frequency and timing of the texts were tailored based on information collected from participants during two scripted, telephone tailoring interviews conducted by CC delivery-staff.

**Table 1:** HLaC+Txt Intervention components

Intervention component	Content
**Initial tailoring interview**	Participants worked with the CC delivery staff to choose: - 1 weight goal (optional) • weight maintenance or weight loss (1–6 kg/12wks) [30, 31] - 1 or 2 SMART goals (specific, measurable, achievable, realistic, time-based) targeting diet and/orphysical activity behaviour • details for each SMART goal, including: • 2 preparatory behaviours • 2 barriers to reaching the goal • 2 solutions to overcome these barriers - preferred frequency (# per fortnight) and timing (time of day) of texts
**12-week tailoring interview**	Participants worked with the CC delivery staff to discuss: - the acceptability/usefulness of text frequency/timing/content - the option to adjust initial tailoring interview responses
**Text messages (see** Table 2**)**	Based on tailoring interview responses, participants received: - 1–11 texts/fortnight for 24 weeks

#### Tailoring interviews

Based on available CC resources, the person delivering the tailoring interviews at each CC was either the CC HLaC coach (i.e. nurse or allied health professional), or CC research assistant who had conducted the pre- and post-HLaC program assessments. All CC delivery staff were trained by a researcher to deliver HLaC+Txt via telephone-delivered training session/s (approximately 30 min in length) and were emailed a Training Manual which outlined HLaC+Txt and how to conduct the tailoring interviews.

The initial tailoring interview was conducted with each participant at baseline HLaC+Txt (after completion of HLaC) and repeated approximately 12 weeks later. Key behaviour change techniques incorporated into the tailoring interview scripted conversations were problem solving and relapse prevention which are challenging to do via text messaging and are more easily targeted through conversation [33, 34]. At the 12-week tailoring interview participants’ text message response rates (over the first 12 weeks of the program) were prefilled in the script to give the CC delivery-staff context of how often the participant was interacting with the texts. Feedback questions were included to facilitate participants’ thoughts on their experiences with the text messages over the previous 12 weeks to encourage the choice of appropriate content for the tailoring variables for the remainder of the intervention.

#### HLaC+txt text messages

HLaC+Txt text messages reinforced strategies based on behaviour change techniques (BCT) discussed by the HLaC coaches during the initial 6-month telephone coaching program which support weight, and diet and physical activity behaviour maintenance (e.g. monitoring of weight, diet and physical activity behavior, problem solving and goal setting) [35–37]. These BCT were targeted across six different types of texts with a total of 245 different texts available to send during the program (Table 2). Minimal abbreviations were used in the text messages (i.e., to ‘2’; you ‘u’; your ‘ur’; for ‘4’). A researcher (JJ) was responsible for monitoring any text message replies from participants, and the CC, therefore, requested that the text messages were signed off using the researcher’s name (not the HLaC coach’s name).

**Table 2:** Examples of each type of HLaC+Txt text message and frequency of delivery

Text Message Type	BCT^**a**^ targeted [32]	Example text message	Frequency
**Goal checks for physical activity and/or diet**	Goal setting (behaviour)	How are u going Barry? Able to eat salad for lunch 5x this week? Text me back so I know how ur going. Jenny	Weekly or fortnightly
**Goal check replies** (only sent if a participant replied to a physical activity and/or diet goal check)	Feedback on behaviour	Well done! U have made so much progress over the last 12 months Barry. Pause & reflect on ur achievements, noticed u feel better? Jenny	Sent if a participant responded to a goal check message (24 different replies for Yes AND 24 different replies for No.
	Problem solving Social support	Think about what got in the way Barry. If it was being too busy then try talking to ur partner. Jenny	
	Self-reward (unspecified)	Great news Barry. Well done! Take time 2 enjoy ur reward & give urself a pat on the back - u really deserve it! Jenny	
	Self-monitoring of behaviour	A good way 2 get back on track Barry is 2 keep a diary of ur diet 4 a few days. Refer to your diary when needed. Jenny	
	Goal setting (behaviour)	Barry that’s fantastic! I don’t want u 2 get bored with walking 5x/wk. so if u need a change try a different exercise goal this week. Jenny	
**Behavioural prompts for physical activity and/or diet**	Prompts/cues Self-monitoring of behaviour	Written down ur exercise lately Barry? Keep a record of when u walk 30 min 5 days this week. Jenny	1–2/week per behaviour (optional) -participants could choose the days to suit habitual timing of their behaviour.
	Problem solving	Remember 2 eat 5 serves veges/day. If being too busy is stopping u try 2 use frozen veges. Jenny	
	Habit formation	Barry u wanted 2 go to the gym 3x this week. Make sure u put out ur exercise gear 2 make it easier. Jenny	
**Weight self-monitoring prompt**	Prompt/cue Self-monitoring of outcome of behaviour	Time 2 weigh yourself Barry. It’s good to note ur progress in ur HLaC weight tracker. Jenny	Fortnightly (optional)
**Goal setting reminder for weight**	Goal setting (outcome)	It’s important to re-set ur weight goal Barry. U currently want to lose 2 kg. If u have a new goal for the next 6 weeks, reply & let me know. Jenny	At 6wks & 18wks (optional)
**Goal setting reminder for physical activity and/or diet**	Review behaviour goal(s) Goal setting (behaviour)	Need to update ur goal to walk 30 min 6 x/week? If u have a new goal for the next 6 weeks, reply & let me know. Jenny	At 6wks & 18wks

^a^*BCT* Behaviour change technique

Text messages, limited to 160 characters, were automatically generated and sent using a researcher-developed platform (www.propelo.com.au). Data from the tailoring interviews were imported from the database into the platform by a researcher, enabling individually tailored texts to be pre-programmed and sent, tailored to schedules for each participant. This system was programmed to automatically recognise participant goal check “yes” or “no” replies and trigger automatic responses (goal check replies). However, if participants replied with any other text (in addition to or instead of “yes” or “no”) an email was sent to the researcher, who would trigger the appropriate goal check response or tailor a response in cases when the goal check response frameworks were not appropriate. Participants were able to modify their weight or behavioural goals or their text message tailoring preferences (e.g., timing, frequency) by replying to goal check texts and these replies were also emailed automatically to the researcher, who made the appropriate adjustments to the tailoring data. If the participant reported any health or medical issues, the participant was asked via text if they wished to adjust their goals, and corresponding changes to the tailoring data were made. Participants were able to opt out of the program at any time by replying “STOP” to any text.

### Control cohort treatment

This cohort of participants received no further contact following the completion of the HLaC coaching calls, except from the researchers to conduct the post-HLaC+Txt trial assessment and to receive the written feedback from this assessment.

### Data collection

Data collected and assessment tools for each of the RE-AIM dimensions are reported in Table 3. The RE-AIM dimensions are reported in chronological order (i.e. adoption, reach, implementation, effectiveness, maintenance) rather than the acronym order.

**Table 3:** RE-AIM evaluation indicators of the HLaC+Txt extended contact intervention

Dimension	Indicator	Collection method/assessment tool
**Adoption**	Uptake of HLaC+Txt by CCs	• Number of CCs approached ^**a**^ • Number of CCs that declined & reasons ^**a**^
	Staff delivering HLaC+Txt	• Qualifications of CC staff delivering the intervention ^**a**^
	Adjustments/adaptations/barriers for each CC	• Documentation of telephone/email interactions with CC staff ^**a**^ • Qualitative interviews with CC staff conducted by a researcher (JJ)
**Reach**	Uptake by HLaC completers & comparison between CCs	• Number of participants approached ^**b**^ • Number of participants deemed ineligible ^**b**^ • Participation rate for those eligible ^**b**^ • Number, timing and reasons for participant withdrawals/ graduations ^**a,b**^ • Program completion rates ^**a,b**^ • A comparison between CCs of all of the above ^**a**^
	Characteristics of HLaC+Txt cohort	Data collected via telephone at the baseline HLaC assessment [27] • Demographic/health characteristics of participants (control/intervention) & those who declined ^**b**^ • Comparison of the characteristics between these three cohorts ^**a**^ • Comparison of the characteristics of those who participated with datasets of national cancer survivors (Australian Institute of Health and Welfare, 2017) to examine representativeness ^**a**^
**Implementation**	Intervention delivery	• Completion rates and duration of initial and 12-week tailoring interviews ^**b**^ • Number and type of text messages sent to each participant ^**c**^ • Number of prompted/unprompted text message replies from participants ^**c**^ • Number of replies to participant text messages that required the researcher to edit the response ^**c**^ • Participant withdrawal/ graduation rates and average intervention length ^**a,b**^ • Number/modality (text, telephone, or email) of requests received during the intervention to change goals/text preferences or hold texts ^**a,b**^ • A comparison across CCs of all of the above ^**a**^ • Qualitative interviews with CC staff conducted by a researcher (JJ).
	Cost of delivery	• Number of CC staff who delivered the intervention ^**a,b**^ • Cost (AUD$): staff time ^**a,b**^ & sending text messages ^**a**^
**Effectiveness**	Anthropometric, physical activity, dietary outcomes	Self-reported during the HLaC+Txt trial pre- ^**b**^ & post-program ^**a**^ telephone assessments, for control & intervention cohorts • Weight; waist circumference; MVPA (Australian Institute of Health Welfare, 2003); vegetable & fruit intake (Reeves et al., 2015), fat & fibre behaviour^d^ (Rutishauser et al., 2001)
	Quality of Life^e^ (Sanderson et al., 2002)	Self-reported during the HLaC+Txt trial pre- ^**b**^ and post-program ^**a**^ telephone assessments, for control & intervention cohorts
	Participant satisfaction with HLaC+Txt program	• At the HLaC+Txt trial post-program assessment all participants rated overall satisfaction with/usefulness of the texts for meeting goals on a five-point scale^**a**^ • Qualitative interviews with a sample of participants by a researcher (JJ).
	Unintended consequences	• 12-week tailoring interview ^**b**^ • Documentation of text message, telephone, or email interactions/ satisfaction survey & qualitative interviews with participants at HLaC+Txt trial post-program assessment ^**a**^
**Maintenance (individual)**	Anthropometric, physical activity, dietary outcomes	Self-reported during the HLaC+Txt trial follow-up assessment for intervention cohort ^**a**^ • Weight; waist circumference; MVPA; vegetable & fruit intake, fat & fibre behavior^**a,d**^
	Quality of Life ^**e**^	• Assessed during HLaC+Txt trial follow-up assessment for intervention cohort.
**Maintenance (setting)**	Intervention continuation	• Documentation & description of processes. ^**a**^

Documented by: ^**a**^ researcher (JJ), ^**b**^ CC. ^**c**^ Automatically recorded via the propelo™ platform. ^**d**^ Fat & Fibre behaviour scores 1–5, with higher values indicating healthier habits. ^e^Higher values indicate better quality of life

Participant outcome data were collected via telephone at the HLaC+Txt trial pre-program assessment (HLaC post-program assessment) and HLaC+Txt trial post-program assessment (completion of HLaC+Txt for the intervention cohort and 6-months post HLaC completion for control cohort - see Fig. 1) [27, 28]. Maintenance participant outcome data were assessed in the HLaC+Txt follow-up survey following 6-months of no-contact for the HLaC+Txt intervention cohort (see Fig. 1). CC staff conducted the HLaC+Txt trial pre-program assessments and the first author (JJ) conducted the HLaC+Txt trial post-program and follow-up assessments [27, 28]. Assessors were not blinded to the participant’s treatment. All participants were given the option of receiving a written summary of the dietary and physical activity feedback from the assessments, by email or post.

All participants were asked to rate overall satisfaction with and usefulness of the texts for meeting goals on a 5-point scale from “not at all” to “extremely” useful/satisfied, and to provide feedback on the program in an open-ended question.

Semi-structured, in-depth qualitative interviews were conducted with the CC delivery staff during and at the completion of the intervention via telephone (Additional File 1: Staff Interview Guide). In addition, a random sample of participants were interviewed at the HLaC+Txt trial post-program assessment to gather data on their experience with the HLaC+Txt intervention & how the program fit with longer-term cancer care (Additional File 2: Participant Interview Guide). Participants interviewed included those who had completed the entire intervention & those who had withdrawn from intervention prior to completion. Interviews continued until saturation of themes occurred. All qualitative interviews were conducted by a researcher (JJ) via telephone using a semi-structured interview technique, recorded and transcribed verbatim using F4 software (audiotranskription.de, Marburg, Germany). Idiosyncrasies (e.g., um, ahh) and word repetitions were then removed from the transcripts and the data were coded, categorised and themes identified independently by the interviewer (JJ) and another author (BF) using an inductive approach [38, 39]. These two authors then discussed identified themes and agreed on common themes, which were supported by example quotes from the interview scripts.

The cost (AUD$) of delivery of the intervention was calculated from the cost for staff time (for delivering tailoring interviews, training, entering tailoring data into software and manually triggering goal check replies), and the cost of sending text messages. Staff time for delivery of the tailoring interviews was tracked in real-time in the CC databases [27, 28] and the remainder of the staff time for intervention delivery tasks was tracked manually by a researcher (JJ).

#### Sample size

The target sample size for HLaC+Txt was a total of 204 participants (*n* = 102 controls, n = 102 HLaC+Txt participants). Sample size calculations used standard deviations and correlations derived from outcomes of an interim analysis on the first 314 participants from the initial HLaC intervention. This sample size provided 80% power to detect differences between the control and intervention cohorts of: 1.0 kg body weight assuming a standard deviation of 18.0 kg and a pre-post correlation of 0.99. A sample size of 102 for each cohort was also adequate (power ≥ 80%) to detect a difference between the control and intervention cohort in changes of 0.4 serves of fruit per day (SD = 1.09 serves/day, r = 0.41), 0.7 serves of vegetables per day (SD = 1.8 serves/day, r = 0.43), a score of 0.2 on the fat score (SD = 0.51 points, r = 0.67), a score of 0.2 on the fibre score (SD = 0.50 points, r = 0.63), 80 min of MVPA/week (SD = 235 min, r = 0.62), 2.6 cm waist circumference (SD = 16.43 cm, r = 0.92), 4 units mental quality of life (SD = 9.8, r = 0.50) and 4 units physical quality of life (SD = 10.5, r = 0.48).

### Data analysis

**Fig. 2:**
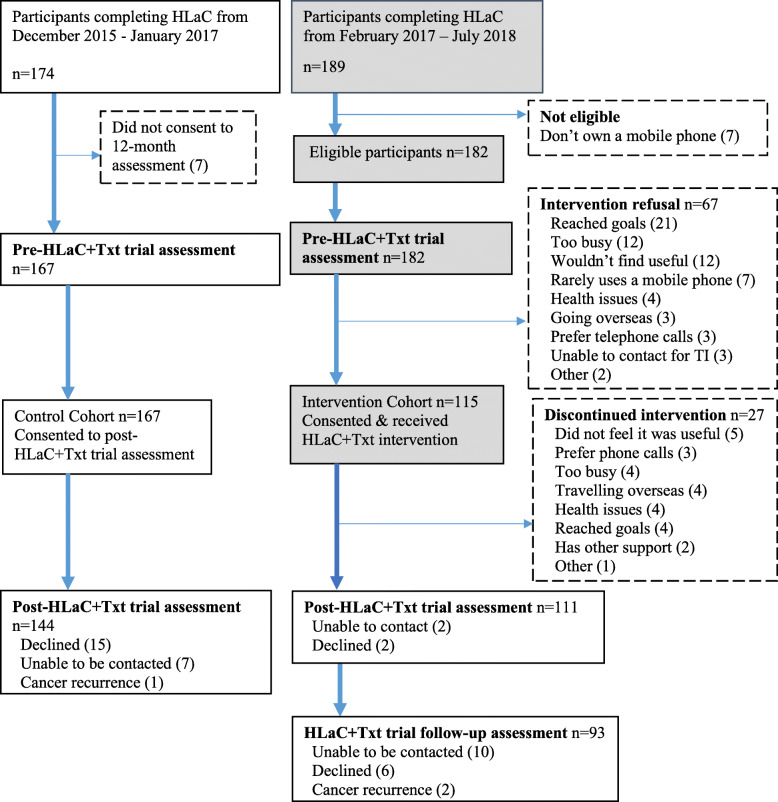
Flow chart of participants in the HLaC+Txt trial

The participant flow through the trial was described (Fig. 2). Reach (participation rates for those eligible for HLaC+Txt) was compared between CCs (referred to as CC1–4 to de-identify) by Chi-square test (*p* < 0.05). Effectiveness data were analysed according to control or intervention cohort regardless of the intervention received [40] (excluding those with missing data at post- and follow-up HLaC+Txt trial assessments). Differences between the two cohorts in HLaC baseline data were compared by t-test (p < 0.05) to identify potential confounding variables. Estimated mean changes in outcomes within groups from baseline to 6-month (HLaC+Txt trial pre- to post-survey) and the effect of intervention (HLaC+Txt less control cohort) were assessed using linear regression models (*p* < .05). Within group maintenance was assessed for the HLaC+Txt intervention group (i.e. estimated mean change between post- and follow-up- surveys) (p < .05). Analysis was adjusted for baseline (HLaC+Txt) values of the outcome, CC and gender (regardless of significance), and other potential confounders that were significant as determined via backwards elimination (*p* < 0.20) (Additional File 3). Maintenance was also adjusted for years since cancer diagnosis (regardless of significance). For context, outcomes at baseline HLaC (mean, SD) and changes during HLaC (pre- to post-program assessment) (paired t-test: unadjusted, *p* < 0.05) are presented for the control and intervention cohorts. A sensitivity analysis with imputed data for missing post-program and follow-up assessment values was conducted using chained equations [41] in SPSS with the adjusted models to test the sensitivity of the conclusions to missing data (m = 20 imputations, except m = 25 for FFBQ fibre post- and follow-up assessment values and for mental quality of life follow-up assessment values). Analysis was performed using SPSS Statistics version 22 (IBM Corp. Armonl, NY), Stata version 13 (StataCorp. Texas, USA), SAS version 9.4 (SAS Institute Inc. NC, USA).

#### Interpretation of findings

The aim of extended contact interventions is for participants to maintain or improve outcomes, and therefore no change within the intervention cohort would be interpreted as a positive finding. Therefore, it was only when findings were statistically significant that cohorts were claimed to have on average “worsened” or “improved” for within cohort changes or claimed to be “better” or “worse” than controls for between-cohort differences. Non-significant findings can indicate either no change/difference in outcome or an insufficient sample size to show a conclusive finding. Therefore, we only described outcomes as “maintained” or cohorts as being “similar” when the finding was both non-significant and the likely true effect size for the change/difference (as seen by the 95% confidence interval) was less than the minimum difference of interest (MDI) [23]. MDI’s were: weight 1.0 kg; waist circumference 1.0 cm; MVPA 30 min; fruit and vegetables 0.5 serves/day; FFBQ fat and fibre index scores 0.2 units; physical and mental quality of life 1.0 unit.

## Results

### Adoption outcomes

All four CCs delivering the HLaC program were approached and agreed to deliver the HLaC+Txt intervention (CC Victoria, CC South Australia, CC New South Wales and CC Western Australia). The CCs each made their own decisions regarding which staff would conduct the tailoring interviews, with two CCs using the HLaC telephone coaches who had been trained in motivational interviewing and two using research assistants who had no motivational interviewing training. These decisions were based on the resources available at each CC.

### Reach outcomes

Of the participants approached to join HLaC+Txt across the four CCs (*n* = 189), 96% (*n* = 182) were eligible and 64% of these (*n* = 115) consented to participate (Fig. 2). There were statistically significant differences in reach across CCs (Chi Square *p* = .004) ranging from 47% in CC4 to 80% in CC1 (Additional File 4). Of the 115 participants who commenced the HLaC+Txt intervention, 88 (77%) completed the intervention. The overall retention rate for assessments from pre- to post-HLaC+Txt trial assessments was 89% (316/356) (intervention cohort 97%, 111/115; control cohort 86%, 144/167). The retention rate for the follow-up assessment after 6-months no-contact for the HLaC+Txt intervention cohort was 81%, 93/115.

Participants in the HLaC+Txt trial (*n* = 282) were mostly female (*n* = 253/90%) survivors of breast cancer (*n* = 181, 64%), who were on average 1.9 years (SD ±3.0) since diagnosis and had a mean age of 58.3 (SD ± 10.9) years and at the pre-HLaC assessment, had a mean BMI of 27.7 kg/m^2^ (SD ± 5.7). Those in the control cohort (*n* = 167) were largely similar to those in the HLaC+Txt intervention cohort (*n* = 115) (Table 4), but had a significantly higher intake of vegetables (serves/day) (*p* < .05) at the pre-HLaC+Txt trial assessment and received a slightly lower (yet statistically significant) number of intervention calls (10.0, SD ±1.6) during the HLaC program than the intervention cohort (10.6, SD ±1.5) (Additional File 5). Those who declined intervention participation (*n* = 67) and those in the control cohort had lower scores for symptom interference than the intervention cohort and a lower number of intervention calls during the HLaC program, and those who declined intervention participation had lower scores for fat intake than the intervention cohort (Additional Files 5 and 6). When compared with the cancer survivor population in Australia [42] the trial participants (*n* = 282) were more likely to be female (90% v 44%, *p* < .001), and the females were more likely to have breast cancer (72% v 36%, *p* < .001) and the males more likely to have lymphoma (21% v 5%, *p* < .001) (Additional File 7).

**Table 4:** Baseline health and demographic characteristics (at Pre-HLaC trial assessment) of HLaC+Txt trial participants

	HLaC+Txt Intervention cohort (*n* = 115)	Control cohort (*n* = 167)
	Mean (SD) or n (%)	
Age (years)	57.5 (10.4)	58.9 (11.2)
Gender (% female)	105 (91.3)	148 (88.6)
CC^a^ enrolled in (%)		
CC1	40 (34.8)	52 (31.1)
CC2	31 (27.0)	48 (28.7)
CC3	19 (16.5)	32 (19.2)
CC4	25 (21.7)	35 (21.0)
Referral source (% from within CC)	67 (58.3)	108 (64.7)
Live in major city (% yes)	92 (80.0)	119 (72.1)
Caucasian (% yes)	102 (89.5)	158 (94.6)
Education (% post school qualifications)	90 (78.3)	136 (81.4)
Employed (% yes)	54 (47.0)	82 (49.1)
Married/ living together (% yes)	75 (65.2)	115 (68.9)
Cancer diagnosis (%)		
Breast	79 (68.7)	102 (61.1)
Lymphoma	7 (6.1)	18 (10.8)
Colorectal	10 (8.7)	14 (8.4)
Prostrate	2 (1.7)	11 (6.6)
Other	17 (14.8)	22 (13.2)
Years since cancer diagnosis	1.76 (2.36)	2.05 (3.3)
Treatment		
Surgery	102 (88.7)	142 (85.0)
Radiotherapy	67 (58.3)	98 (58.7)
Chemotherapy	81 (70.4)	110 (65.9)
Mean number of comorbidities	2.2 (1.8)	2.08 (1.7)
Mental health issue (% depression &/or anxiety &/or nervous disorder)	52 (45)	71 (42.5)
Smoking (% ever smoked)	39 (33.9)	61 (36.5)

^a^*CC* Cancer Council

### Implementation outcomes

#### Staff training

All 16 staff (100%) who delivered the tailoring interviews attended the first 30-min telephone-delivered tailoring interview training session and four staff (25%) attended the second 12-week tailoring interview training session (the two HLaC telephone coaches with motivational interviewing training and two research assistants who had no motivational interviewing training).

#### Intervention delivery

The mean number of weeks participants received the text message intervention ranged from 18.5–22.2 weeks across CCs (mean 21.1, SD = 1.7) (Additional File 8) with some CC adapting the 24-week intervention based on their perceptions at the 12-week tailoring interview of participants’ needs. Participants received a mean of 83 text messages over the length of the intervention ranging from 40 for CC4 to 112 for CC1. The mean (±SD) call duration of the initial tailoring interview was 25.9 (±15.1) minutes and the second tailoring interview was 20.0 (±11.4) minutes. Of the 115 participants who completed the first tailoring interview, 97 (84%) completed the second, ranging from 82 to 91% across CCs. Participants did not receive the second tailoring interview if they withdrew or discontinued from the intervention prior to the second tailoring interview, they were unable to be contacted, or a decision was made at the second tailoring interview that they did not wish to continue with the intervention (early graduation). Withdrawal/graduation rates varied across CCs from 18% (CC1) to 48% (CC4) (Additional File 4). The percent of goal checks participants responded to was a median (25th, 75th percentile) of 70% (50.0, 85.0) and a median (25th, 75th percentile) of 30% (10.0, 55.6) required a goal check response to be triggered by the researcher. Sixty five percent of participants (*n* = 75) replied to the goal re-set texts sent at weeks 6 and 18. At the 12-week tailoring interview 84% (*n* = 97) of participants changed their preference for text message content, frequency and/or timing. The median (25th, 75th percentile) number of texts per fortnight chosen by particpants at the first tailoring interview was 8.2 (7.0, 11.0) and at the 12-week tailoring 6.1 (3.0, 9.0). Of the 115 participants in the program, 25 put the text-messages on hold for between one and 7 weeks for holidays (*n* = 21) or illness (*n* = 4). No participant changed the timing or frequency of the texts via a text message to the coach, apart from 10 participants notifying via text message that they no longer wished to receive the texts.

#### Qualitative interviews with staff

Interviews were conducted with seven delivery staff (two health coaches and five research assistants) (Additional File 9). A key theme was that staff perceptions about implementation aligned with differences in the qualifications of the staff conducting the intervention (i.e. CC HLaC telephone coaches versus CC research assistants). The HLaC coaches felt the tailoring interview flowed well from discussions with participants during the telephone-coaching program, about maintaining lifestyle behaviour changes, *“it helps guide.... their goals going forward”.* In contrast, the CC research assistants identified that their lack of coaching contact with participants meant that they had greater challenges with delivering the tailoring interviews. The qualitative interviews also highlighted differences between the coaches in their support for participant graduation from the intervention at the 12-week tailoring interview. One coach reported that participants *“on a whole”* were keen to receive the full 6 months of text messages as per the intervention protocol, whereas another coach graduated some participants at the 12-week interview *“I think three months is the limit, I think that beyond that I don’t know that they need it as much”*.

#### Participant satisfaction with program

At the post-HLaC+Txt trial assessment most participants were ‘satisfied’ or ‘extremely satisfied’ (77%, 90/110) with the text message program and found the texts ‘useful’ or ‘extremely useful’ for supporting them to meet their behaviour goals (68%, 75/110). Qualitative interviews were conducted with 28 participants (Additional File 10). Participants perceived that HLaC+Txt provided: reminders for maintaining their diet and physical activity behaviours established during HLaC and provided a continuing connection with the program. For some, the switch of coach from the HLaC telephone coach to the researcher who signed off the texts caused a loss of accountability to the program. A common theme throughout the qualitative interviews with participants were reported personal stressors or barriers to achieving their diet, physical activity and weight goals, such as: social issues (employment, finances, family); ongoing treatment side effects and cancer-related symptoms (disturbed sleep, fatigue, cravings, taste changes, mental health issues and joint pain); and chronic conditions such as arthritis.

#### Cost of delivery

A researcher spent a total of 8.5 h training 16 CC staff in the delivery of the HLaC+Txt tailoring interviews. CC delivery staff spent an average of 30 min per participant preparing for and delivering the initial tailoring interview (*n* = 115) and 28 min per participant for the second tailoring interview (*n* = 95). A researcher spent an average of 25 min per participant to enter the data from the first tailoring interview into the text messaging platform and 15 min per participant to enter the second tailoring interview data. A researcher spent an average of one minute/response (*n* = 613) to manually trigger replies to goal checks which were not automatically recognised by the platform. A total of 9502 text messages were sent during the intervention at a cost of $AUD0.15 per text message for a total of $AUD1425.30 (an average of $AUD12.39 per participant). Staff time was costed at $43.85/h. The total cost per participant for delivering HLaC+Txt was on average $AUD85.00.

#### Effectiveness outcomes

##### Within-cohort changes

Both HLaC+Txt and control cohorts had significantly worsened outcomes for body weight, MVPA, vegetable intake, and fat and fibre index scores between the pre- and post-HLaC+Txt trial assessments (Table 5). The intervention cohort maintained fruit intake while fruit intake worsened for the control cohort. Whilst there was no significant change in waist circumference for the intervention cohort, and physical and mental quality of life outcomes for both cohorts, the confidence intervals for these changes were greater than the MDI and the results were therefore inconclusive.

**Table 5:** Anthropometric/behavioural data: HLaC+Txt intervention and control cohorts: baseline HLaC^#^, change from: HLaC pre- to post-survey^#^, HLaC+Txt trial pre- to post-survey, HLaC+Txt trial post- to follow-up-survey and HLaC+Txt trial intervention effects

	Intervention		Control		Intervention effect (HLaC + Txt – control)
	n	Mean change (95% CI)^a^	n	Mean change (95% CI)^a^	Mean difference (95% CI)^b^
**Weight (kg)**					
*HLaC*					
*Pre survey/ baseline - Mean (SD)*	115	77.90 (16.46)	167	78.03 (18.93)	
*Change pre- to post-survey*	114	**−2.13 (−2.91, −1.35)** ^4^	167	**−2.56 (−3.13, − 2.00)** ^4^	
**HLaC + Txt**					
Change pre- to post-survey	111	**1.10 (0.57, 1.63)** ^**1**^	**142**	**1.19 (0.59, 1.79)** ^**1**^	0.09 (−0.71, 0.89)^5^
Change post- to follow-up-survey	93	0.12 (−0.48, 0.73) ^2^		NR	
**Waist circumference (cm)**					
*HLaC*					
*Pre survey/ baseline - Mean (SD)*	115	97.12 (13.84)	167	96.58 (14.96)	
*Change pre- to post-survey*	113	**−5.01 (−6.33, −3.70)** ^4^	165	**−4.35 (−5.38, − 3.32)** ^4^	
**HLaC + Txt**					
Change pre- to post-survey	107	0.68 (−0.36, 1.72)^3^	**144**	**0.97 (0.07, 1.86)** ^**1**^	−0.29 (−1.68, 1.10)^3^
Change post- to follow-up-survey	88	0.01 (−1.08, 1.10)^3^		NR	
**Physical activity Moderate-vigorous (min/week)**					
*HLaC*					
*Pre survey/ baseline - Mean (SD)*	115	209.67 (202.62)	167	207.95 (222.03)	
*Change pre- to post-survey*	115	**164.87 (106.87, 222.87)** ^4^	167	**135.16 (100.79, 169.54)** ^4^	
**HLaC + Txt**					
Change pre- to post-survey	111	**−90.80 (− 128.17, −53.43)**^**1**^	**144**	**−84.62 (− 117.39, − 51.86)**^**1**^	−6.20 (− 56.14, 43.78)^3^
Change post- to follow-up-survey	93	−6.87 (−47.09, 33.36) ^3^	144	NR	
**Fruit (serves/day)**					
*HLaC*					
*Pre survey/ baseline - Mean (SD)*	115	1.79 (1.07)	167	1.80 (1.15)	
*Change pre- to post-survey*	115	**0.20 (0.04, 0.36)** ^4^	167	**0.32 (0.16, 0.49)** ^4^	
**HLaC + Txt**					
Change pre- to post-survey	111	−0.05 (−0.19, 0.09)^2^	142	**−0.14 (−0.26, −0.02)**^**1**^	0.09 (− 0.10, 0.27) ^5^
Change post- to follow-up-survey	93	−0.06 (− 0.22, 0.11)^2^		NR	
**Vegetables (serves/day)**					
*HLaC*					
*Pre survey/ baseline - Mean (SD)*	115	3.18 (1.84)	167	3.26 (1.86)	
*Change pre- to post-survey*	115	**0.66 (0.31, 1.01)** ^4^	167	**1.07 (0.77, 1.38)** ^4^	
**HLaC + Txt**					
Change pre- to post-survey	111	**−0.45 (−0.73, −0.17)**^**1**^	**144**	**−0.47 (− 0.72, − 0.23)**^**1**^	0.02 (− 0.35, 0.40) ^5^
Change post- to follow-up-survey	93	−0.69 (−1.00, − 0.38)^1^		NR	
**FFBQ Fat index (score 0–5)**					
*HLaC*					
*Pre survey/ baseline - Mean (SD)*	113	3.16 (0.53)	167	3.26 (0.49)	
*Change pre- to post-survey*	113	**0.33 (0.24, 0.42)** ^4^	167	**0.32 (0.27, 0.38)** ^4^	
**HLaC + Txt**					
Change pre- to post-survey	108	**−0.14 (−0.20, −0.08)**^**1**^	**142**	**−0.11 (− 0.17, − 0.06)**^**1**^	−0.03 (− 0.12, 0.06) ^5^
Change post- to follow-up-survey	90	−0.04 (− 0.11, 0.03)^2^		NR	
**FFBQ Fibre index (score 0–5)**					
*HLaC*					
*Pre survey/ baseline - Mean (SD)*	115	2.85 (0.54)		2.78 (0.48)	
*Change pre- to post-survey*	103	**0.21 (0.12, 0.30)** ^4^	160	**0.26 (0.18, 0.33)** ^4^	
**HLaC + Txt**					
Change pre- to post-survey	98	**−0.08 (−0.15, −0.00)**^**1**^	**138**	**−0.11 (− 0.17, − 0.06)**^**1**^	0.04 (− 0.05, 0.13)^5^
Change post- to follow-up-survey	81	0.03 (− 0.05, 0.11)^2^		NR	
**Quality of Life Physical (SF-12), 0–100**					
*HLaC*					
*Pre survey/ baseline - Mean (SD)*	115	39.98 (10.89)	167	39.64 (10.04)	
*Change pre- to post-survey*	115	**5.38 (3.38, 7.39)** ^4^	167	**7.19 (5.59, 8.78)** ^4^	
**HLaC + Txt**					
Change pre- to post-survey	111	−0.89 (−2.46, 0.68)^3^	144	−1.12 (−2.49, 0.26)^3^	0.66 (−1.43, 2.75)^3^
Change post- to follow-up-survey	93	−1.32 (−3.14, 0.50)^3^		NR	
**Quality of Life Mental (SF-12), 0–100**					
*HLaC*					
*Pre survey/ baseline - Mean (SD)*	115	48.17 (10.42)	167	49.38 (10.22)	
*Change pre- to post-survey*	115	1.80 (−0.08, 3.68) ^2^	167	**1.94 (0.50, 3.39)** ^4^	
**HLaC + Txt**					
Change pre- to post-survey	111	−1.39 (−2.92, 0.14)^3^	144	−0.48 (− 1.82, 0.86)^3^	−0.91 (− 2.95, 1.14)^3^
Change post- to follow-up-survey	93	− 1.29 (− 2.91, 0.34)^3^		NR	

^#^ Presented for the sub-sample who entered the HLaC+Txt trial for context to interpret the changes during the HLaC+Txt program (unadjusted mean changes estimated by paired t-test within completers)

^a^Mean changes estimated within groups and between group difference using regression models for completers [adjusted for HLaC+Txt pre-survey values of the outcome, CC and gender (regardless of significance), and other confounders that were significant (*p* < 0.02) (Additional File Table 3)]

Statistical significance at *p* < .05. Within cohort: ^1^ Worsened ^2^ Maintained ^3^ Inconclusive ^4^Improved, Between cohort: ^3^ Inconclusive ^5^ Similar NR = not recorded

##### Between-cohort effects

No significant intervention effects were seen for changes between the pre- and post-HLaC+Txt trial assessment in any of the anthropometric, dietary, MVPA or quality of life measures (Table 5). Results, however, were inconclusive for MVPA and quality of life as the confidence intervals included the MDI.

##### Sensitivity analysis

The results of the multiple imputation analyses (Additional File 11) generally supported the main analyses results, except, that due to slightly narrower confidence intervals in the multiple imputation analyses the increase in waist circumference in controls and decrease in physical activity in both cohorts was ‘inconclusive’ (rather than ‘worsened’) as the change was not significant. Fibre intake in the intervention cohort was ‘maintained’ rather than ‘worsened’ and physical quality of life in the control cohort ‘worsened’ rather than being ‘inconclusive’.

#### Maintenance outcomes (participant level)

##### Within-cohort change

The HLaC+Txt cohort maintained outcomes for weight, fruit intake, and fat and fibre index scores between post-HLaC+Txt and follow-up (after 6-months no contact) (Table 5). Results during this same time period were inconclusive for waist circumference, MVPA, physical and mental quality of life outcomes for the intervention cohort as the confidence intervals included the MDI.

##### Sensitivity analysis

The results of the multiple imputation analyses (Additional File 11) supported the main analyses results for changes during 6-months no contact for the HLaC+Txt cohort.

#### Maintenance outcomes (setting level)

To date, the initial HLaC program is being adapted and offered by three CC at a reduced scale or in a web-based format [28]. The CCs are funding the delivery of these adapted programs, as the research grant funding is complete and at the time of publishing this manuscript no CCs were continuing to offer text-message delivered extended contact.

## Discussion

Using the RE-AIM framework, this implementation trial evaluated a text message-delivered, extended contact intervention targeting healthy weight, diet and physical activity, for cancer survivors. Importantly, this program was delivered by the major community-based cancer support, non-profit organisation in Australia. All four CCs adopted the HLaC+Txt intervention and the program was feasible to implement, however reach and implementation results varied greatly across CCs. The intervention was not effective when outcomes were compared to a historical control cohort at the completion of the intervention, although data collected at the HLaC+Txt trial follow-up assessment (6 months after text message completion) suggests that the benefits of the extended contact intervention may have been delayed.

Three key reasons are suggested for the lack of effectiveness findings at the end of the intervention: 1) the variability between CCs in adoption, reach and implementation, 2) the lack of intervention focus on holistic support for the social and mental health requirements of this cohort of cancer survivors, and 3) the disconnect in accountability and rapport between the initial HLaC and the HLaC+Txt programs.

Variations in HLaC+Txt program delivery across the CCs were driven by differences in CC resources leading to variations in the qualifications and experience of the intervention delivery staff [43–45]. Ensuring delivery staff are skilled in motivational interviewing [46], and techniques to provide participant support for coping with barriers to maintaining diet and physical activity [47] may be a key area for consideration with future implementation. Further, a protocol for a shorter intervention delivery period (i.e. 12 weeks) could be explored by comparing the effectiveness of a 12-week and 6-month intervention trial period using a randomized controlled trial.

In addition, as suggested by the qualitative data, the needs of the participants for social and mental health support may not have been met by the behaviour-change focused, text message-delivered program [48]. As well as a previous cancer diagnosis, participants had an average of two co-morbidities, and many reported mental health conditions similar to those seen in cross sectional Australian data of cancer survivors [49–51]. The most frequently reported unmet need of cancer survivors, following treatment completion is support for psychosocial issues [52]. Incorporating the option for mental health support into the text message frameworks and the option for participants to choose mental health goals targeting stress, depression, anxiety and sleep may improve intervention acceptability for this population [53] and have potential for supporting the mental health of cancer survivors. Alternatively, a program that incorporates text messages which are supplemented with additional telephone support for emotional and social well-being may address this deficit and may be a more appropriate extended contact modality for some participants [47, 54, 55]. Furthermore, with recent developments, this support may be feasible through triaging levels of intervention intensity via artificial intelligence [56, 57].

The loss of connection, rapport and accountability established between the coach and client during HLaC, may have further exacerbated the lack of support participants experienced for social and mental health issues. The text messages were signed-off with a researcher’s name (rather than the HLaC coach’s name). Qualitative feedback suggests this influenced the acceptability and effectiveness of the text messages for some participants who had lost the connection with their original HLaC coach and CC. A previous review of extended contact interventions suggested that the contact with the interventionist is a key component of the success of these interventions [16]. Other researchers have hypothesized that established relationships enhance the effectiveness and implementation of lifestyle interventions into practice and reduce attrition [58–60]. The existing program is a way to “step down” the intensity of the relationship and wean the participant on to a more cost-effective means of communication. However, continuity of care between the coach and client may still be required for such programs to be effective.

After 6 months of no contact following the end of HLaC+Txt weight, fruit intake and fat and fibre outcomes were maintained by the intervention cohort. This suggests that the text messages may have influenced participants’ ability to maintain changes in diet and exercise behaviour in the longer term. A similar effect has been reported in breast cancer survivors [19] and adults [61] who received a text message-delivered, extended contact intervention following a lifestyle intervention. The text messages may have promoted longer-term maintenance of self-regulation skills such as self-monitoring and encouraged BCTs including engaging support, goal setting and techniques for forming habits [62, 63]. Alternatively, the improved follow-up outcomes may have been a result of the additional 6 months that participants had to adjust their behaviour in response to the weight gain experienced at the end of extended contact.

### Strengths

Evaluating this extended contact, text message-delivered intervention in a service delivery setting adds to the broader evidence on dissemination and implementation outcomes, where interventions are delivered in real world, rather than optimal research conditions [64]. This research was conducted in partnership with the CCs who took ownership of the program. Participant engagement with the intervention was positive and there was low participant attrition. Although participants were more likely to be female, breast cancer survivors compared to the general survivor population in Australia, the program did reach participants with a range of previous cancer diagnosis types, meeting an identified need in the community [14]. The qualitative feedback added to our understanding of the important components of extended contact interventions for this population, including: the background and experience of the delivery staff; the importance of the continuity in the delivery staff across telephone and text modalities to maintain rapport with participants; and the additional social, health and mental support requirements of this target group [65].

### Limitations

HLaC+Txt was not included in the initial research protocol for HLaC [27] and a historical control study design was used due to timeline restrictions. Participants in the control cohort may have been referred to other services in the community (e.g. ENRICH [66], dietitians) and thus the between-cohort effectiveness findings of HLaC+Txt may have been attenuated. The research design relied on self-report data, as objective measures are a challenge in a program covering four states of Australia and targeting both urban and rural participants. Outcome assessments were completed at 18 months from baseline for the HLaC+Txt intervention cohort, but not the control cohort, due to limited researcher resources.

## Conclusions

Although feasible to deliver and generally well accepted by staff and participants, this implementation study revealed that a text message-delivered extended contact intervention was not effective in the short term at supporting maintenance of behaviour change in a sample of cancer survivors. The benefit of such interventions, however, may be observed longer term. Collaboration with community partners has strengthened our understanding of the core program components that will inform future implementation. Maintaining support for participants based on ongoing relationships with CC coaching staff skilled in motivational interviewing may improve accountability and outcomes. In addition, offering a program that is responsive to participant’s changes in health and social circumstances is likely to improve acceptability.

## Supplementary Information

**Additional file 1.** Qualitative Interview Script - Staff – Completion of HLaC+Txt.

**Additional file 2.** Qualitative Interview Script - Participants – Completion of HLaC+Txt.

**Additional file 3: Table 1.** Confounders considered and adjusted for in main and sensitivity analyses.

**Additional file 4: Table 2.** Rates of participant eligibility, participation, withdrawal/graduation and completion across the four Cancer Councils.

**Additional file 5: Table 3.** Anthropometric and behavioural characteristics (at pre-HLaC+Txt trial assessment) and number of HLaC intervention calls received.

**Additional file 6: Table 4.** Baseline characteristics of HLaC participants who declined the HLaC+Txt trial.

**Additional file 7: Table 5.** Gender, cancer type and age of the Australian cancer survivor population and the HLaC+Txt trial participants.

**Additional file 8: Table 6.** Length of intervention and average text message dose sent and received per participant by Cancer Council.

**Additional file 9: Table 7.** Themes identified from the qualitative feedback from HLaC+Txt intervention staff interviews.

**Additional file 10: Table 8.** Themes identified from the qualitative feedback from HLaC+Txt intervention participants at the interviews.

**Additional file 11: Table 9.** Anthropometric and behavioural outcomes within the HLaC+Txt intervention (*n* = 115) and control cohorts (*n* = 167): from pre- to post-HLaC+Txt trial assessments, post-HLaC+Txt trial to follow-up assessment and HLaC+Txt intervention effects using multiple imputation for missing data.

## Data Availability

The datasets used and/or analysed during the current study are available from the corresponding author on reasonable request.

## Abbreviations

BMI: Body Mass Index
CC: Cancer Council
FFBQ: Fat and Fibre Behaviour Questionnaire
HLaC: Healthy Living after Cancer
HLaC+Txt: Healthy Living after Cancer + Text
MDI: Minimum Difference of Interest
QoL: Quality of Life
RE-AIM: Reach, Effectiveness, Adoption, Implementation, Maintenance
MVPA: Moderate-to-Vigorous Physical Activity
SMART: Specific, Measureable, Achievable, Realistic, Time-based
SD: Standard Deviation
