# Supplementary material for: Evaluation of the Healthy Living after Cancer text message-delivered, extended contact intervention using the RE-AIM framework
Source: BMC Cancer. 2021 Oct 7;21:1081. doi: 10.1186/s12885-021-08806-4 (PMC8496009; doi:10.1186/s12885-021-08806-4)
Supplement: Supplementary file 1 — Additional file 1. Qualitative Interview Script - Staff – Completion of HLaC+Txt. [file 12885_2021_8806_MOESM1_ESM.docx]

Additional File 1: Qualitative Interview Script - Staff – Completion of HLaC+Txt

1. After 18- months of delivery what is your impression of the text message program?
2. What do you feel the particpants impressions of the texting program were?
3. How do you feel about CC… continuing with the text message program in the longer term?
4. What parts of the program do you feel didn’t work or were not acceptable to the particpants? (How did you feel about the length of the TI? The length of the Txt programe) (If we did continue what changes that you feel we could make to improve it?)
5. What parts of the program do you feel worked well or were most acceptable to the particpants?
6. How do you feel the HLaC+Txt program integrates with the overall HLaC program?
7. How do you think the participants felt about the texts being signed off by Jenny? (How important do you feel the coach name is on the text messages?)
8. In what ways, if any, do you feel the text messages helped participants to maintain the diet and exercise changes established during the telephone coaching?
9. Do you feel many of the participants joined HLaC+Txt to help with research rather than to benefit themselves?
10. How did you contact the participants for the TI and TI2?
11. How many times on average did you have to try before your contacted them?
12. When do you schedule TI1 for and when did you do this? (same time as PP assess or at feedback call)
13. What sort of support do you suggest participants seek if they are struggling after HLaC.
14. How did you find using REDCap?
15. I am interested in the background training of the staff – RA versus counsellor?
