# Supplementary material for: Evaluation of the Healthy Living after Cancer text message-delivered, extended contact intervention using the RE-AIM framework
Source: BMC Cancer. 2021 Oct 7;21:1081. doi: 10.1186/s12885-021-08806-4 (PMC8496009; doi:10.1186/s12885-021-08806-4)
Supplement: Supplementary file 2 — Additional file 2. Qualitative Interview Script - Participants – Completion of HLaC+Txt. [file 12885_2021_8806_MOESM2_ESM.docx]

**Additional File 2:** **Qualitative Interview Script – Participants – Completion of HLaC+Txt**

Your feedback will help us improve the service in the future. (You are free to stop this interview at any stage, and you are also free to not answer any questions which you do not feel comfortable answering.) These questions should take about 10 minutes. I would like to audio record our conversation from now on. Do you consent to having this call recorded?

**Reasons for joining the program:**

What motivated you to receive the text messages offered after completing the telephone calls with the Cancer Council?

**Overall experience of the text messages:**

How did you find receiving the text messages over the last 6 months? (Prompt if required: Can you tell me some of the positives and some of the negatives?)

**Maintaining behaviour:**

In what ways, if any, did the text messages help you to maintain the diet and exercise changes you established during the telephone coaching?

**Text goals:**

When you started the text messages with xxxx (CC coach/RA) you set a goal on diet and physical activity (check TI) which a lot of the texts referred to. Can you tell me how you found setting goals for diet and PA?

**Suggestions for changes to texts:**

What sorts of changes would you suggest we make to the texts we send? (Different types, number, timing).

**Managing cancer care:**

Can you tell me how the text messages fitted in with your longer term cancer care?

Can you tell me your thoughts on receiving text messages to support your mental or emotional health?

**Continuing longer term:**

If it was available, how would you feel about continuing to receive texts beyond the 6 months of texts you have already received?

Do you have any other comments or feedback you would like to make on the text messages that you have not already given:

**Thank you for your time and participating in this interview. We appreciate your comments and time. A reminder you are free to contact the Cancer Council at any tie if you have any concerns.**
