# Supplementary material for: Evaluation of the Healthy Living after Cancer text message-delivered, extended contact intervention using the RE-AIM framework
Source: BMC Cancer. 2021 Oct 7;21:1081. doi: 10.1186/s12885-021-08806-4 (PMC8496009; doi:10.1186/s12885-021-08806-4)
Supplement: Supplementary file 3 — Additional file 3: Table 1. Confounders considered and adjusted for in main and sensitivity analyses. [file 12885_2021_8806_MOESM3_ESM.docx]

Additional File 3: Table 1: Confounders considered and adjusted for in main and sensitivity analyses

| **Outcome** | **Adjusted in analyses^a^** |
| --- | --- |
| Weight (kg) | Baseline HLaC+Txt trial weight, gender, CC, major city |
| Waist circumference (cm) | Baseline HLaC+Txt trial waist circumference, gender, CC, distress, MDASI Symptom Interference, change in waist circumference category HLaC |
| Moderate-vigorous physical activity  (min/week) | Baseline HLaC+Txt trial PA, BMI, gender, age, CC, post school education, distress |
| Fruit (serves/day) | Baseline HLaC+Txt trial fruit serves, gender, CC, major city, change in fruit serves category HLaC, comorbidity count |
| Vegetables (serves/day) | Baseline HLaC+Txt trial vegetable serves, gender, CC, change in fruit serves category HLaC |
| FFBQ Fat index (score 0-5) | Baseline HLaC+Txt trial FFBQ fat index score, gender, CC, change in FFBQ fat index category HLaC |
| FFBQ Fibre index (score 0-5) | Baseline HLaC+Txt trial FFBQ fibre index score, gender, age, CC, change in FFBQ fibre index category HLaC, post-school education |
| Quality of Life - Physical (SF-12), 0-100 | Baseline HLaC+Txt trial physical QoL, gender, CC, change in physical QoL category HLaC, comorbidity count, MDASI Symptom Interference, BMI baseline HLaC+Txt |
| Quality of Life - Mental (SF-12), 0-100 | Baseline HLaC+Txt trial mental QoL, gender, CC, change in mental QoL category HLaC, distress |

^a^Models adjusted for baseline (pre-HLaC+Txt trial assessment) values of the outcome, CC and gender (regardless of significance), and other potential confounders that were significant at p<0.2 selected from the following: From baseline HLaC - CC, gender, age major city (yes/no), post-school education (yes/no), breast cancer (yes/no), comorbidity count. From baseline HLaC+Txt trial - distress category (no distress/distress -no impact/distressed -impacts) and MDASI Symptom Interference. Change in outcome category <MDI/>=MDI (baseline to end of HLaC). BMI omitted from weight and waist circumference models as these adjust for weight and waist circumference.
