# Supplementary material for: Evaluation of the Healthy Living after Cancer text message-delivered, extended contact intervention using the RE-AIM framework
Source: BMC Cancer. 2021 Oct 7;21:1081. doi: 10.1186/s12885-021-08806-4 (PMC8496009; doi:10.1186/s12885-021-08806-4)
Supplement: Supplementary file 4 — Additional file 4: Table 2. Rates of participant eligibility, participation, withdrawal/graduation and completion across the four Cancer Councils. [file 12885_2021_8806_MOESM4_ESM.docx]

Additional File 4: Table 2: Rates of participant eligibility, participation, withdrawal/graduation and completion across the four Cancer Councils

|  | CC1 | CC2 | CC3 | CC4 | Total |
| --- | --- | --- | --- | --- | --- |
| Number of participants eligible (n) | 50 | 32 | 46 | 54 | 182 |
| Number of participants deemed ineligible (n, %) | 4 (8) | 0 (0) | 1 (2) | 2 (4) | 7 (4) |
| Participation numbers and rates for those eligible (n, %)^#^ | 40 (80) | 19 (59) | 31 (67) | 25 (47) | 115 (64) |
| Withdrawal/graduation from intervention  Numbers and rates* (n, %)   - prior to tailoring interview 2 - at tailoring interview 2 - after tailoring interview 2 | 7 (18)  3  2  2 | 4 (21)  1  1  2 | 4 (13)  3  0  1 | 12 (48)  0  10  2 | 27 (24) |
| Intervention completion (received 24 weeks text messages)^+^ - number and rates (n, %) | 31 (78) | 14 (74) | 26 (84) | 13 (52) | 84 (73) |

^#^ Statistically significant difference in participation rates across CCs (p=.004) based on Chi square test.

^*^ Statistically significant difference in withdrawal rates across CCs (p=.007) based on Chi square test combining CC1, CC2, CC3 (due to small cell sizes) to compare with CC4.

^+^n=4 participants did not withdraw but only received 23 weeks of text messages
