# Supplementary material for: Evaluation of the Healthy Living after Cancer text message-delivered, extended contact intervention using the RE-AIM framework
Source: BMC Cancer. 2021 Oct 7;21:1081. doi: 10.1186/s12885-021-08806-4 (PMC8496009; doi:10.1186/s12885-021-08806-4)
Supplement: Supplementary file 5 — Additional file 5: Table 3. Anthropometric and behavioural characteristics (at pre-HLaC+Txt trial assessment) and number of HLaC intervention calls received. [file 12885_2021_8806_MOESM5_ESM.docx]

Additional File 5: Table 3: Anthropometric and behavioural characteristics (at pre-HLaC+Txt trial assessment) and number of HLaC intervention calls received

|  | HLaC+Txt Intervention cohort  (n=115) | Control  Cohort  (n=167) | Declined Intervention  cohort  (n=67) |
| --- | --- | --- | --- |
|  | Mean (SD) or n(%) | | |
| BMI (kg/m^2^)  Weight (kg)  Waist circumference (cm)  MVPA (minutes/week)  Dietary behaviours  Vegetables (servings/day)  Fruit (servings/day)  FFBQ Fat index (score 1-5)  FFBQ Fibre index (score 1-5)  Mental quality of life (SF-12), 0-100  Physical quality of life (SF-12), 0-100  Mean symptoms severity (MDASI) 0-10,  Mean symptoms interference (MDASI) 0-10  Fear of cancer recurrence (CARQ-4), 0-40  Level of distress impact  Distress impact  Alcohol (number of drinks/week) | 27.9 (5.4)  75.6 (15.7)  92.1 (13.9)  374.5 (311.7)  3.8 (1.8)*  2.0 (0.9)  3.5 (0.5) ^  3.1 (0.5)  50.0 (10.4)  45.4 (10.4)  3.1 (1.8)  2.7 (2.4)*^  12.5 (8.4)  2.0 (2.4)  1.4 (2.3)  2.0 (3.9) | 27.6 (5.9)  75.5 (17.5)  92.1 (13.7)  343.1 (254.6)  4.3 (2.0)*  2.1 (0.9)  3.6 (0.5)  3.0 (0.5)  51.3 (9.0)  46.8 (10.0)  3.0 (1.9)  2.1 (2.1)*  12.6 (9.2)  2.0 (2.3)  1.1 (1.9)  3.1 (5.4) | 28.4 (6.8)  77.7 (17.9)  95.6 (14.4)  317 2 (240.9)  3.7 (1.4)  2.1 (0.8)  3.4 (0.4)^  3.0 (0.5)  50.3 (10.8)  45.9 (10.5)  2.6 (1.8)  2.0 (2.1)^  11.4 (9.6)  2.0 (2.6)  1.5 (2.6)  3.3 (7.2) |
| Number of HLaC intervention calls received | 10.6 (1.5)^*^^ | 10.0 (1.6)^*^ | 9.49 (1.9)^ |

^*^statistically significant difference between cohort (HLaC intervention consent and control cohorts) (p<0.05) based on t-test
^statistically significant difference between cohort (HLaC intervention consent and decline cohorts) (p<0.05) based on t-test
