# Supplementary material for: Evaluation of the Healthy Living after Cancer text message-delivered, extended contact intervention using the RE-AIM framework
Source: BMC Cancer. 2021 Oct 7;21:1081. doi: 10.1186/s12885-021-08806-4 (PMC8496009; doi:10.1186/s12885-021-08806-4)
Supplement: Supplementary file 6 — Additional file 6: Table 4. Baseline characteristics of HLaC participants who declined the HLaC+Txt trial. [file 12885_2021_8806_MOESM6_ESM.docx]

Additional File 6: Table 4: Baseline characteristics of HLaC participants who declined the HLaC+Txt trial

| Health and demographic characteristics | Declined Intervention (n=67)  Mean (SD) or n(%) |
| --- | --- |
| Age (years)  Gender (% female)  State enrolled in CC (%)  CC1  CC2  CC3  CC4  Referral source (%CC) Major city (% yes)  Caucasian (% yes)  Education (% post-school)  Employed (% yes)  Married/ living together (% yes)  Cancer diagnosis (%)  Breast   Lymphoma  Colorectal  Prostrate Other  Years since diagnosis  Treatment  Surgery  Radiotherapy  Chemotherapy  Mean number of comorbidities  Mental health issue (% depression &/or anxiety &/or nervous disorder)  Smoking (% ever smoked) | 60.3 (12.7)  56 (83.6)  10 (14.9)  15 (22.4)  13 (19.4)  29 (43.3)  50 (74.6)  49 (75.4)  63 (94.0)  50 (74.6)  32 (47.8)  39 (58.2)  41 (61.2)  7 (10.4)  4 (6.0)  2 (3.0)  13 (19.4)  1.7 (1.7)  58 (86.6)  47 (70.1)  46 (68.7)  2.3 (1.7)  27 (40)  28 (41.8) |
