# Supplementary material for: Evaluation of the Healthy Living after Cancer text message-delivered, extended contact intervention using the RE-AIM framework
Source: BMC Cancer. 2021 Oct 7;21:1081. doi: 10.1186/s12885-021-08806-4 (PMC8496009; doi:10.1186/s12885-021-08806-4)
Supplement: Supplementary file 7 — Additional file 7: Table 5. Gender, cancer type and age of the Australian cancer survivor population and the HLaC+Txt trial participants. [file 12885_2021_8806_MOESM7_ESM.docx]

Additional File 7: Table 5: Gender, cancer type and age of the Australian cancer survivor population and the HLaC+Txt trial participants

|  | Diagnosed with cancer in the previous 5 years (2012)^*^ | HLaC+Txt  (Txt & control cohorts,)  (n=282) | HLaC+Txt – intervention declined cohort (n=67) |
| --- | --- | --- | --- |
| Female | 44%^a^ | 90%^a^ | 84% |
| Males  - Prostrate cancer - Colorectal cancer - Lymphoma | 41% 13% 5%^b^ | 45% 7% 21%^b^ | 18% 9% 36% |
| Females - Breast cancer - Colorectal cancer - Ovarian/cervical | 36%^a^ 13% 4% | 72%^a^ 9% 5% | 73% 5% 4% |
| Age - 20- 29 - 30 – 39 - 40 - 49 - 50 - 59 - 60 - 69 - 70 - 79 - 80+ | 1% 3% 8% 18% 29% 25% 16% | 1% 3% 19% 33% 29% 12% 3% | 3% 6% 9% 30% 30% 19% 3% |

^a^p<.001, ^b^p<.05 (compared using Chi square test)

^*^(Australian Institute of Health and Welfare, 2019b)
