# Supplementary material for: Evaluation of the Healthy Living after Cancer text message-delivered, extended contact intervention using the RE-AIM framework
Source: BMC Cancer. 2021 Oct 7;21:1081. doi: 10.1186/s12885-021-08806-4 (PMC8496009; doi:10.1186/s12885-021-08806-4)
Supplement: Supplementary file 8 — Additional file 8: Table 6. Length of intervention and average text message dose sent and received per participant by Cancer Council. [file 12885_2021_8806_MOESM8_ESM.docx]

Additional File 8: Table 6: Length of intervention and average text message dose sent and received per participant by Cancer Council

|  | **Cancer Council** | | | | **Total** |
| --- | --- | --- | --- | --- | --- |
|  | **CC1 (n=40)** | **CC2 (n=19)** | **CC3 (n=31)** | **CC4 (n=25)** | **n=115** |
|  |  | Mean (range) | | | |
| **Length of intervention (weeks) - Mean (range)** | 22.2  (6 – 24) | 22.1  (2-24) | 21.6  (4-24) | 18.5  (11-24) | 21.1  (2-24) |
|  |  | Median (25^th^, 75^th^ percentile) | | | |
| **Text dose sent**  **(total)** | 112 (84, 130) | 72 (62, 83) | 103 (65, 130) | 40 (25, 55) | 83 (48, 119) |
| **Text dose sent (weekly)*** | 4.7 (3.8, 5.5) | 3.0 (2.6, 4.0) | 4.7 (3.2, 5.5) | 2.0 (1.6, 3.6) | 3.7 (2.8, 5.1) |
| **Dose diet and/or physical activity goal checks sent** | 20 (18, 20) | 17 (10, 20) | 19 (16, 20) | 13 (10, 17) | 19 (12, 20) |
| **Dose diet and/or physical activity behaviour prompts sent** | 76 (50, 94) | 47 (35, 54) | 72 (35, 94) | 22 (4,27) | 48 (24, 84) |
| **Goal reset sent** | 6 (4,6) | 4 (3,5) | 6 (4,6) | 3 (2,5) | 5 (3,6) |
| **Recognised responses to goal checks (i.e. yes or no replies)** | 6 (2, 11) | 4 (1, 11) | 8 (2, 15) | 7 (2, 10) | 6 (2, 11) |
| **Unrecognised responses to goal checks** | 4 (2,11) | 5 (1,10) | 3 (1,6) | 5 (2,7) | 4 (2,9) |
| **Goal reset replies** | 1 (0,3) | 1 (0,2) | 1 (0,3) | 1 (0,2) | 1. (0,2) |

*Calculation based on total text dose and number of weeks in the program. Significant variation by state by ANOVA (p<,001)
