# Supplementary material for: Evaluation of the Healthy Living after Cancer text message-delivered, extended contact intervention using the RE-AIM framework
Source: BMC Cancer. 2021 Oct 7;21:1081. doi: 10.1186/s12885-021-08806-4 (PMC8496009; doi:10.1186/s12885-021-08806-4)
Supplement: Supplementary file 9 — Additional file 9: Table 7. Themes identified from the qualitative feedback from HLaC+Txt intervention staff interviews. [file 12885_2021_8806_MOESM9_ESM.docx]

Additional File 9: Table 7: Themes identified from the qualitative feedback from HLaC+Txt intervention staff interviews

| Differences between the coaches and the RAs, in their experience of delivering the tailoring interview | During the tailoring interview the coaches, who had established rapport with the participants during the telephone coaching program, could reflect on *“some of the barriers during the program …the solutions that did work, so drawing back on the things that the participants have had success with”.* In contrast, the RA’s identified that their lack of coaching contact with particpants meant that they found challenges with delivering the tailoring interview. Although the tailoring interview was scripted, they did not have knowledge of the participant’s successes and challenges during the telephone coaching program and were less familiar with the terminology used during the program e.g. goals, barriers and solutions. They felt that the knowledge of the telephone coaches would be helpful when conducting the tailoring interviews. *“I don’t do any of the calls throughout the 6-months… I’m not sure what they’ve (the participants) struggled with”* and *“I think maybe we weren’t the right people to do the phone calls or we just didn’t have the knowlegde”* . For some RA’s, the tailoring interview script helped overcome the problems they encoutered with lack of experience with the program and participants: *“the prompting was really … helpful, having the ideas, like ‘take your lunch to work’ or ‘plan your meals’”.* However, *o*thers found that they initially lacked familiarity with the terminology used in the tailoring interviews and that the role became easier with practice: *“some of the terminology was not all that clear to me at times.. in talking of ....goals and actions”* which improved with experience *“it is just getting practice”*. |
| --- | --- |
| Perceptions of the ideal length of the intervention varied for different coaches | One coach found “*when I’ve called them at the 3-month mark they were all really glad it was another 3 months, on a whole …., because they were finding it helpful, it was giving them that nudge” whereas another coach reported that “some people… mentally need that extra support and the 6-months is the right time for them”*, however some participants were graduated from the program at the second tailoring interview *“I think three months is the limit, I think that beyond that I don’t know that they need it as much” and “it is such an individual thing. I’ve got to listen to them (the participants)”.* |
