# Supplementary material for: Evaluation of the Healthy Living after Cancer text message-delivered, extended contact intervention using the RE-AIM framework
Source: BMC Cancer. 2021 Oct 7;21:1081. doi: 10.1186/s12885-021-08806-4 (PMC8496009; doi:10.1186/s12885-021-08806-4)
Supplement: Supplementary file 10 — Additional file 10: Table 8. Themes identified from the qualitative feedback from HLaC+Txt intervention participants at the interviews. [file 12885_2021_8806_MOESM10_ESM.docx]

Additional File 10: Table 8: Themes identified from the qualitative feedback from HLaC+Txt intervention participant interviews

| **Participants perceived that HLaC+Txt provided reminders for maintaining their diet and physical activity behaviours established during the HLaC telephone coaching.** | *“The text program topped it all off, because I think that had I not followed up with the texts I think that I might have slipped back”* and *“it was still like that lifeline that I was getting* (started crying and got very emotional) *sorry I got all emotional …. so you think, oh don’t give up, don’t give up”.* |
| --- | --- |
| **For some, the connection and accountability of HLaC was lost** | The text message program *“lacked personal support”* and was *“too automated”.* Participants understood the step down from the telephone coaching was required but some preferred the telephone conversations, which provided more accountability than the texts. The participants had no established relationship with ‘Jenny’ (the text message ‘coach’), *“I didn't know whether Jenny was a real person or automated so didn't have the same rapport and didn't feel personally responsible to her”* and the texts *“felt automated, impersonal. I don't think I ever wanted to do the texts in the beginning I joined the text program for the research.”* |
| **Social, mental health and chronic disease issues created barriers** | Suggestions for incorporating text messages that supported mental health, including anxiety, were common *“a whole level of emotional/mental level that could be addressed as well possibly, ‘have you done something you enjoy today?’, the mental well-being and emotional stability you do really struggle with”.* For some the text messages offered a medium for providing ongoing support through these issues *“Very grateful for it. Particularly because the stuff I receive at the moment is very negative - about divorce, work, kids (problems), the texts are very positive”.* However, text messages are not as adaptable as telephone conversations when health issues are challenging *“when you are not well. It was like she wasn’t listening – why would I would want to set a new goal when I couldn’t do it, that was a bit annoying”.* Whereas for others it came at just the right time *“For me personally I had an injury from soccer in the month or two before the text messages started, I was about to give up on exercise, the text messages were motivation to keep doing it for my health. It gives you motivation and a reminder.”* |
| **Negative/ unintended consequences** | Early feedback from participants identified that they did not like to be reminded of their ‘cancer’ in the texts so the abbreviation *HLaC+Txt* was used rather than *Healthy Living after Cancer*. During the qualitative interviews, some participants identified issues with the frequency (*“too many”*) and timing (*“I probably set the time for receiving the messages at the wrong time“*) of the text messages |
