# Supplementary material for: Evaluation of the Healthy Living after Cancer text message-delivered, extended contact intervention using the RE-AIM framework
Source: BMC Cancer. 2021 Oct 7;21:1081. doi: 10.1186/s12885-021-08806-4 (PMC8496009; doi:10.1186/s12885-021-08806-4)
Supplement: Supplementary file 11 — Additional file 11: Table 9. Anthropometric and behavioural outcomes within the HLaC+Txt intervention (n = 115) and control cohorts (n = 167): from pre- to post-HLaC+Txt trial assessments, post-HLaC+Txt trial to follow-up assessment and HLaC+Txt intervention effects using multiple imputation for missing data. [file 12885_2021_8806_MOESM11_ESM.docx]

Additional File 11: Table 9: Anthropometric and behavioural outcomes within the HLaC+Txt intervention (n=115) and control cohorts (n=167): from pre- to post-HLaC+Txt trial, post-HLaC+Txt trial to follow-up assessment and HLaC+Txt trial intervention effects using multiple imputation for missing data

| **HLaC+Txt: Change** | **Intervention** | **Control** | **Intervention effect**  **(HLaC+Txt – control)** |
| --- | --- | --- | --- |
|  | Mean change (95% CI)^a^ | Mean change (95% CI)^a^ | Mean difference (95% CI)^b^ |
| **Weight (kg)** |  |  |  |
| Pre- to post-HLaC+Txt assessment | **1.18 (0.60, 1.75)** ^1^ | **1.15 (0.66, 1.64)** ^1^ | 0.03 (-0.72, 0.77)^4^ |
| Post-HLaC+Txt to follow-up assessment | 0.26 (0.31, 1.00) ^2^ |  |  |
| **Waist circumference (cm)** |  |  |  |
| Pre- to post-HLaC+Txt assessment | 0.61 (-1.46, 1.67) ^3^ | 0.85 (-0.05, 1.75) ^3^ | -0.25 (-1.62, 1.13)^3^ |
| Post-HLaC+Txt to follow-up assessment | -0.04 (-1.14, 1.07) ^3^ |  |  |
| **Physical activity** Moderate-vigorous (min/week) |  |  |  |
| Pre- to post-HLaC+Txt assessment | -87.34 (-110.10, -49.00) ^3^ | -79.98 (-123.70, -50.98) ^3^ | -7.361 (-54.92, 40.22)^3^ |
| Post-HLaC+Txt to follow-up assessment | 0.50 (-38.12, 39.12) ^3^ |  |  |
| **Fruit (serves/day)** |  |  |  |
| Pre- to post-HLaC+Txt assessment | -0.06 (0.20, 0.08) ^2^ | **-0.15 (-0.27, -0.03)** ^1^ | 0.09 (-0.10, 0.28) ^4^ |
| Post-HLaC+Txt to follow-up assessment | -0.05 (-0.22, 0.11) ^2^ |  |  |
| **Vegetables (serves/day)** |  |  |  |
| Pre- to post-HLaC+Txt assessment | **-0.39 (-0.67, -0.11)** ^1^ | **-0.43 (-0.67, -0.18)** ^1^ | 0.04 (-0.34, 0.41) ^4^ |
| Post-HLaC+Txt to follow-up assessment | **-0.65 (-0.95, -0.35)** ^1^ |  |  |
| **FFBQ Fat index (score 0-5)** |  |  |  |
| Pre- to post-HLaC+Txt assessment | **-0.14 (-0.21, -0.08)** ^1^ | **-0.11 (-0.16, -0.05)** ^1^ | -0.03 (-0.12, 0.05) ^4^ |
| Post-HLaC+Txt to follow-up assessment | -0.05 (-0.12, 0.01) ^2^ |  |  |
| **FFBQ Fibre index (score 0-5)** |  |  |  |
| Pre- to post-HLaC+Txt assessment | -0.07 (-0.14, 0.00) ^2^ | **-0.11 (-0.17, -0.05)** ^1^ | 0.04 (-0.05, 0.13) ^4^ |
| Post-HLaC+Txt to follow-up assessment | 0.03 (-0.05, 0.12) ^2^ |  |  |
| **Physical Quality of Life (SF-12), 0-100** |  |  |  |
| Pre- to post-HLaC+Txt assessment | -0.86 (-2.43, 0.71) ^3^ | **-1.54 (-2.90, -0.18)** ^1^ | 0.66 (-1.43, 2.75) ^3^ |
| Post-HLaC+Txt to follow-up assessment | -0.86 (-2.43, 0.71) ^3^ |  |  |
| **Mental Quality of Life (SF-12), 0-100** |  |  |  |
| Pre- to post-HLaC+Txt assessment | -1.42 (-2.93, 0.09) ^3^ | -0.42 (-1.71, 0.88) ^3^ | -1.00 (-3.00, 1.00) ^3^ |
| Post-HLaC+Txt to follow-up assessment | -1.63 (-3.31, 0.06) ^3^ |  |  |

^a^Mean changes estimated within groups and between group difference using linear regression models [adjusted for pre-HLaC+Txt trial assessment values of the outcome, CC and gender (regardless of significance), and other confounders that were significant (p<0.02) (Supplemental Table 3)].
^c^Bold indicates statistical significance at p<.05. ^1^ Worsened ^2^ Maintained ^3^ Inconclusive ^4^ Similar
